# A comparative analysis of mRNA enrichment strategies and guidance for improving their efficiency

**DOI:** 10.1038/s41598-025-02082-z

**Published:** 2025-05-23

**Authors:** Angelika Andrzejewska-Romanowska, Ewa Tykwińska, Paweł Śledziński, Katarzyna Pachulska-Wieczorek

**Affiliations:** Department of RNA Structure and Function, Institute of Bioorganic Chemistry, Polish Academy of Sciences, Poznan, Poland

**Keywords:** mRNA enrichment, Ribosomal RNA depletion, Yeast transcriptome, RNA, Transcriptomics, Isolation, separation and purification

## Abstract

The pervasive presence of ribosomal RNA (rRNA) in total RNA poses a considerable challenge to the accurate analysis of cellular transcriptomes. In this study, we comprehensively analyze strategies for *Saccharomyces cerevisiae* mRNA enrichment, aiming to either separate polyadenylated RNAs or selectively remove rRNAs. Our findings reveal that a single round of mRNA enrichment in recommended conditions proves insufficient for both methods, prompting the exploration of strategies to enhance their efficiency. We show that adjusting the oligo (dT) magnetic beads-to-RNA ratio leads to significant improvement, but even better results can be achieved with two rounds of mRNA enrichment. We propose experimental conditions that reduce rRNA content in total yeast RNA to less than 10%, as confirmed by capillary electrophoresis and NG sequencing. Based on the obtained data, we selected the most time- and cost-effective option of polyadenylated RNA selection in yeast total RNA. Furthermore, we demonstrate that RNA modification with SHAPE reagent (NAI) does not interfere with the optimized mRNA enrichment protocol. These insights contribute to mRNA enrichment strategies and underscore the importance of optimizing mRNA isolation methodologies for downstream analyses.

**Supplementary Information:**

The online version contains supplementary material available at 10.1038/s41598-025-02082-z.

## Introduction

The high abundance of rRNA molecules in total RNA samples poses a significant challenge in RNA-based studies. In eukaryotes, rRNA accounts for most of the total RNA content, often comprising over 80% or more of the transcriptome^1–3^. Precisely, in common baker’s yeast *Saccharomyces cerevisiae*, rRNA constitutes approximately 70–80% of the total RNA content, whereas tRNA and mRNA comprise only 15% and 5%, respectively^4^. In other eukaryotes, the rRNA content in total RNA is very similar, as demonstrated, for example, in HEK293 cells (~ 85%), rat liver (~ 80%), human kidney (~ 85%), human pancreas (~ 85%) or *Trypanosoma brucei* (~ 85%)^5–8^. The problem of rRNA contamination becomes apparent during downstream transcriptome analyses. The overwhelming presence of rRNA can bias sequencing results by dominating the read counts^9^. This bias reduces the sensitivity and accuracy of differential gene expression analysis, leading to misinterpretation of experimental results^7^. Moreover, rRNA can hinder the detection and analysis of other RNA species, including low-abundance transcripts such as non-coding RNAs (ncRNAs), which are particularly interesting in many research areas^10^. Caused by rRNA contamination, low sequencing depth of non-rRNA species can also induce difficulties for their secondary structure mapping, using strategies coupled with NGS, such as SHAPE-seq, SHAPE-MaP, or DMS-seq^11^.

Diverse methods have been developed to mitigate the impact of rRNA contamination and improve the accuracy of downstream analyses. They aim to capture the population of polyadenylated RNAs or to selectively remove rRNA molecules while preserving the integrity and representation of other RNA species. Oligo (dT)-based methods rely on the hybridization of the poly(A) tails of RNA molecules to oligo (dT) probes attached to magnetic beads. It allows for selective capture and enrichment of eukaryotic polyadenylated RNA species, excluding most RNA molecules that lack poly(A) tails, such as most rRNAs^12,13^. These techniques are straightforward and relatively simple to implement but limited to polyadenylated transcripts and, therefore, are not suitable for capturing non-polyadenylated RNA species, such as most ncRNAs. On the other hand, if poly(A) RNAs are the aim of the study, these methods significantly enrich a pool of full-length transcripts. Targeted rRNA-depletion methods are typically based on the application of oligonucleotides complementary to rRNAs to separate or degrade them in total RNA samples^14^. The specifically hybridized probes can be physically extracted together with rRNA using streptavidin-covered magnetic beads or can be recognized and cleaved by duplex-specific nucleases. An essential limitation of targeted rRNA depletion is the necessity of application species-specific probes. Moreover, the effectiveness of targeted rRNA depletion may be reduced since probes used in commercially available kits may only partially cover rRNA sequences or do not target 5S rRNA at all^14^.

Here, we comprehensively analyzed mRNA enrichment strategies for *Saccharomyces cerevisiae* total RNA. We compared the efficiency of the targeted rRNA depletion (RiboMinus™ Transcriptome Isolation Kit from Invitrogen) and poly(A) RNA selection (Poly(A)Purist™ MAG Kit from Invitrogen, and Oligo (dT)_25_ Magnetic Beads from New England Biolabs). Our data demonstrate that following a single round of mRNA enrichment under recommended conditions, rRNA still makes up roughly 50% of the total yeast RNA content, regardless of the method utilized. Therefore, we explored strategies to enhance mRNA enrichment efficiency. It involved testing different ratios of oligo (dT) magnetic beads to RNA and implementing a pre-treatment step, resulting in two rounds of mRNA enrichment. As a result, we present improved experimental conditions that reduce rRNA content in total yeast RNA to less than 10%. We also show that in vivo RNA modification with 2-methylnicotinic acid imidazolide (NAI), a widely used SHAPE reagent, does not interfere with the mRNA enrichment protocol developed in our study. Additionally, we demonstrate that the TapeStation electrophoresis can be successfully used for rRNA content assignment as an attractive and cheaper alternative to NG sequencing.

## Results

### Optimizing mRNA enrichment: one round is insufficient

To establish the most efficient way for mRNA enrichment in yeast total RNA samples, we evaluated two different commercially available reagent sets: The RiboMinus™ Transcriptome Isolation Kit from Invitrogen (in subsequent sections referred to as RiboMinus kit or RM) and the Poly(A)Purist™ MAG Kit from Invitrogen (referred to as MAG kit or MAG) (Table 1). Their efficiency was compared to the Oligo (dT)_25_ Magnetic Beads from New England Biolabs (referred to as (dT)_25_ beads or (dT)_25_) that are provided without ready-to-use buffers, requiring the user to prepare all the necessary solutions. The RiboMinus kit employs locked nucleic acids (LNA) oligonucleotides and magnetic beads to selectively bind and remove yeast 18S and 25S rRNA molecules, enriching the non-rRNA fraction. The MAG kit and (dT)_25_ beads are designed to collect mRNA by exploiting the poly(A) tail recognition, thereby reducing non-poly(A) RNA content.

**Table 1:** Comparison of the mRNA enrichment methods used in the study.

	RiboMinus™ Transcriptome Isolation Kit (Invitrogen)	Poly(A)Purist™ MAG Kit (Invitrogen)	Oligo (dT)_25_ Magnetic Beads (New England Biolabs)
Magnetic beads modification	Covered with streptavidin (+5′ biotinylated LNA oligos)	Covered with oligo (dT)	Covered with oligo (dT)
Target RNA	18S and 25S yeast rRNA	poly(A) RNA	poly(A) RNA
Beads regeneration	NO	NO	YES (up to two times)
Reagents included	All buffers included	All buffers included	All buffers must be prepared
Time required per one round (without precipitation)	45 min	60 min-90 min	40 min
Recommended RNA amount and volume	2–10 µg; < 20 µl	30 µg-8 mg; ≥ 100 µl	75 µg; 100 µl*
Recommended beads-to-RNA ratio	3000 µg/10 µg (300:1)	75 µg/75 µg (1:1)	1000 µg/75 µg (13.3:1)* 75 µg/75 µg (1:1)**
Price (2024); bead amount	674.00 USD (36 mg in 3 ml)	921.65 USD (8 mg in 0.8 ml)	356.00 USD (25 mg in 5 ml)
Price per 10 µg of RNA input	56.17 USD	1.15 USD	1.90 USD* (0.63 USD when beads are used three times) 0.14 USD** (0.05 USD when beads are used three times)

*Ratio recommended by Green and Sambrook^15^.

**Ratio recommended in this work.

We followed the manufacturers’ protocols for RiboMinus and MAG kits. As recommended, we used 10 µg of total yeast RNA per single purification reaction with the RiboMinus kit (300:1 beads-to-RNA ratio). 75 µg of total yeast RNA was applied for a single reaction with the MAG kit, and the beads-to-RNA ratio was 1:1. According to the manufacturer, the MAG kit allows such a low ratio without efficiency loss due to the improved buffer recipe. For the (dT)_25_ beads, the manufacturer’s protocol was designed for direct mRNA isolation from mammalian cells that lack a cell wall, and it combines cell lysis and mRNA binding steps. Therefore, to enrich mRNA from an already isolated total yeast RNA sample, we followed the protocol of Green and Sambrook, including the recommended 13.3:1 beads-to-RNA ratio^15^. To evaluate the efficacy of applied mRNA enrichment strategies, we quantified the 18S and 25S rRNA content in total yeast RNA using the TapeStation capillary electrophoresis system. This system provides a high-resolution separation of RNA fragments based on their size, allowing for the detection and quantitative analysis of distinct RNA populations. We quantified the electrophoretic bands corresponding to rRNA using the ImageQuantTL 10.2 software by measuring the areas under the peaks. The obtained data were presented as a percentage of the remaining rRNA, calculated relative to the total RNA content in the sample.

**Fig. 1:**
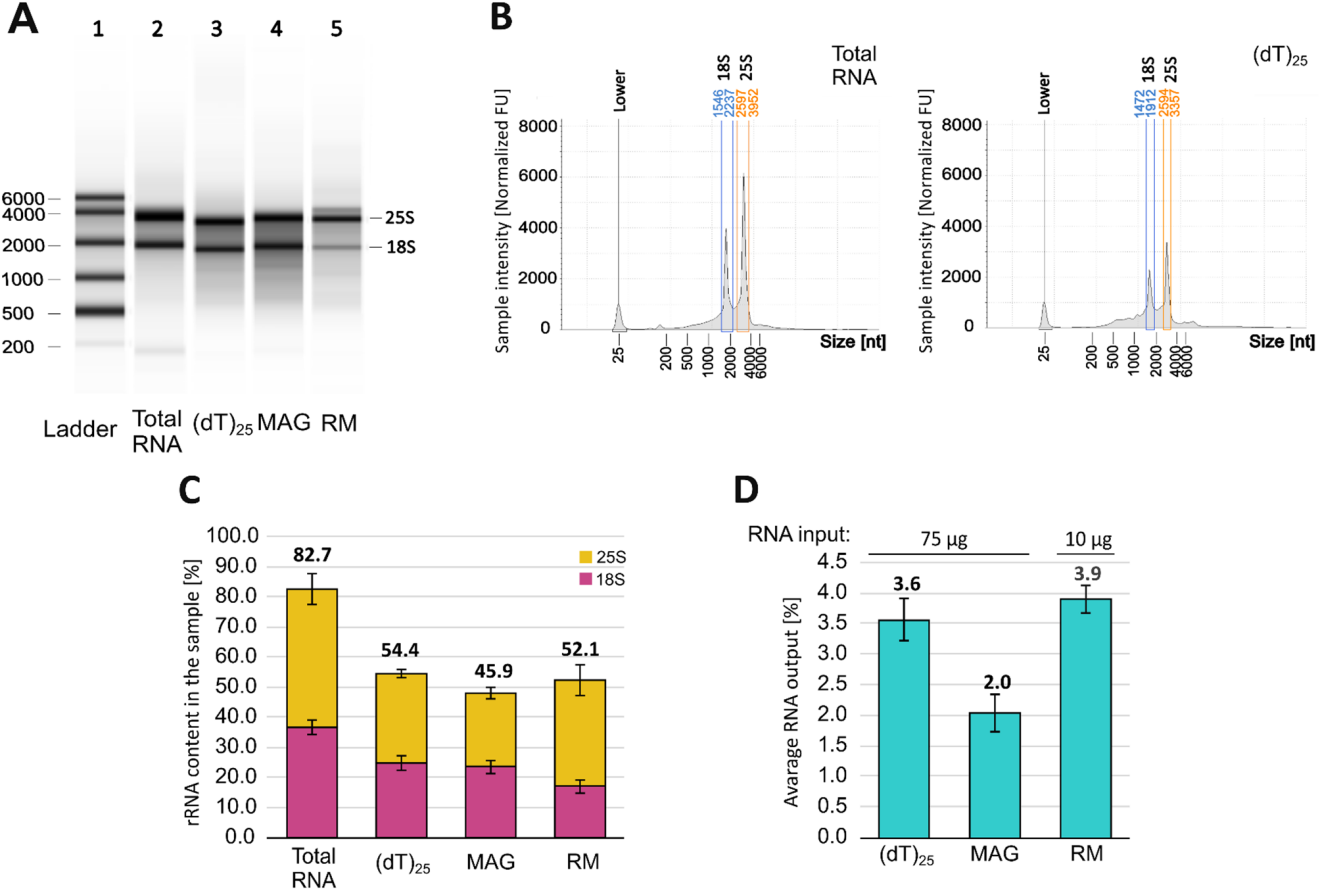
Comparative analysis of yeast mRNA enrichment efficiency using diverse methods. (**A**) The electrophoretic results from the TapeStation platform. Lanes represent (1) RNA ladder; (2) total RNA input; (3–5) RNA samples after mRNA enrichment. (**B**) The representative electropherograms from the TapeStation platform. The electropherogram results for the other approaches are presented in Supplementary Fig. 1. (**C**) Bar plot summary of rRNA content in the output samples. (**D**) Bar plot summary of the average output of RNA presented as a percentage of the input. The RNA input for each approach is shown above the corresponding bar.

Upon analyzing the input total RNA sample, two leading bands or peaks representing the 18S and 25S rRNAs were detected on electropherograms (Fig. 1A,B). The total RNA population consisted mainly of rRNA (82.7%), with 18S rRNA accounting for over 36% and 25S rRNA around 46% (Fig. 1C). For all tested approaches of mRNA enrichment, we detected a decrease in the intensity of 18S and 25S rRNA signals (Fig. 1A,B, Supplementary Fig. 1). Nevertheless, rRNA still accounted for approximately 50% of the RNA in the output samples, showing low efficacy of all tested procedures (Fig. 1C). The MAG kit and (dT)_25_ beads depleted comparable portions of both rRNAs, with the MAG kit demonstrating a slightly higher 25S rRNA removal. The RiboMinus kit primarily reduced the 18S rRNA content, with a weaker reduction observed for the 25S rRNA. The average RNA output ranged from 2% to 3.9% of the total RNA input, with the MAG kit exhibiting the lowest yield (Fig. 1D). The Qubit assessment of RNA integrity showed that none of the applied methods caused RNA degradation and all output RNA samples exhibited a very good quality (RNA IQ score above 8.5).

### Optimizing mRNA enrichment: manipulation of the beads-to-RNA ratio

**Fig. 2:**
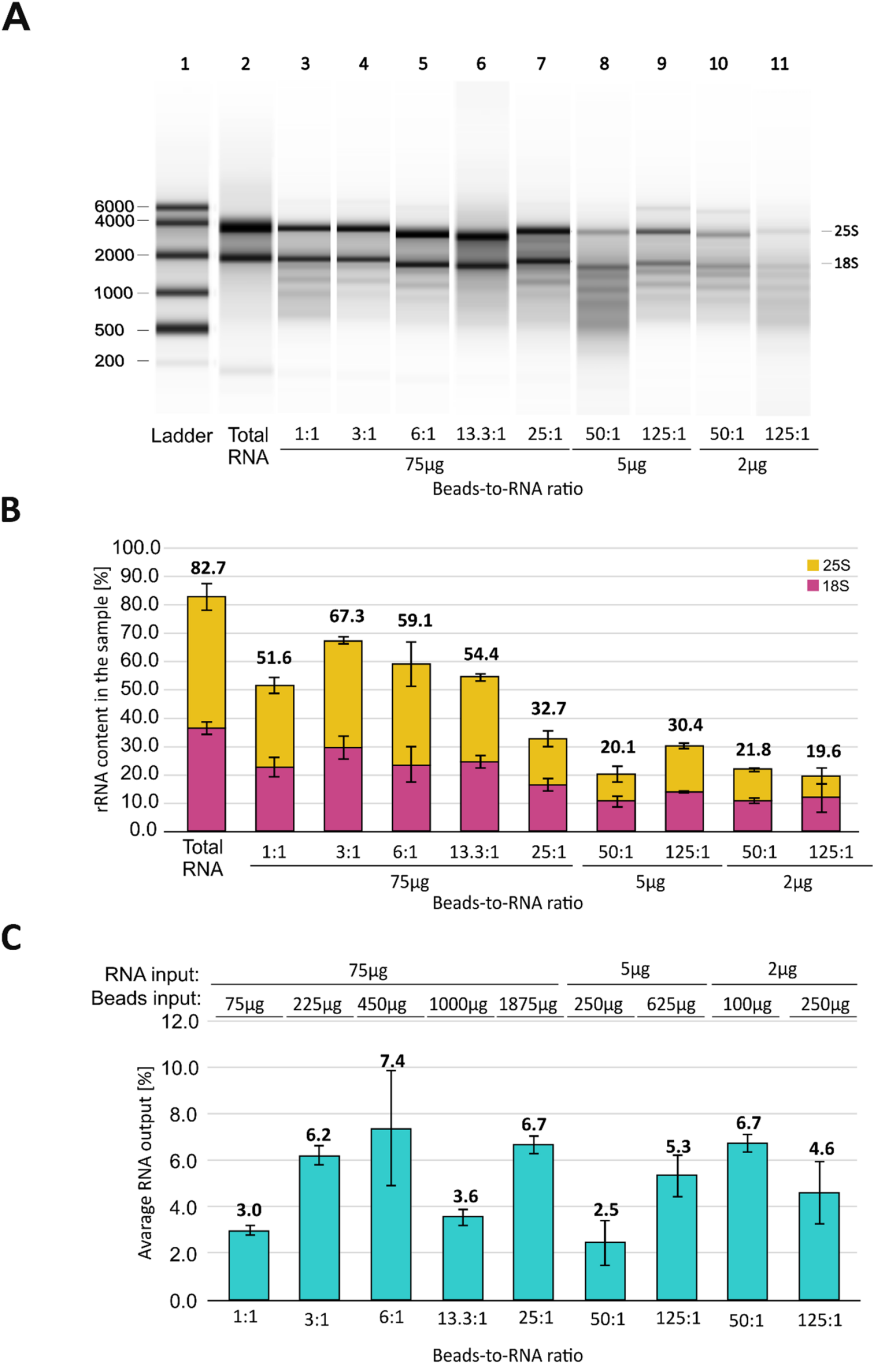
Results of mRNA enrichment using different (dT)_25_ beads-to-RNA ratios. (**A**) The electrophoretic results from the TapeStation platform for different ratios. Lanes represent (1) RNA ladder; (2) total RNA sample; (3–11) RNA samples after mRNA enrichment using different ratios and RNA inputs. (**B**) Bar plot summary of rRNA content in the samples after mRNA enrichment. (**C**) Bar plot summary of average RNA output after mRNA enrichment. The inputs of RNA and beads are shown above the corresponding bars.

To develop a more efficient approach to yeast mRNA enrichment, we used (dT)_25_ beads and searched for the optimal beads-to-RNA ratios (Fig. 2A, B). We found that lowering the ratio from the recommended earlier 13.3:1 to 6:1, or even 1:1, has a negligible impact on the efficiency of mRNA enrichment (54.4% vs. 59.1% and 51.6% of rRNA content, respectively) (Fig. 2B). It showed that applied mRNA enrichment protocol allows using significantly smaller amounts of (dT)_25_ beads without efficiency drop, lowering the cost of the procedure (Table 1). When we increased the beads-to-RNA ratio to 25:1, the rRNA content measured after the mRNA enrichment dropped to 32.7%. The further ratio increase to 50:1 and 125:1 reduced rRNA content to about 20% in RNA output samples. It represents a significant improvement in mRNA enrichment over previously recommended conditions. However, it also necessitates the usage of a substantial quantity of (dT)_25_ beads, which may not be cost-effective. To avoid using the extremely large amounts of magnetic beads, we reduced RNA input from 75 to 5 μg or 2 μg for the highest beads-to-RNA ratios. The percentage of RNA output ranged between 3% to 7.4% for 75 μg and 2.5–6.7% for lower RNA inputs (Fig. 2C). These data clearly showed a lack of correlation between RNA input, mRNA enrichment efficiency, and RNA output.

### Optimizing mRNA enrichment: two rounds for enhanced efficiency

**Fig. 3:**
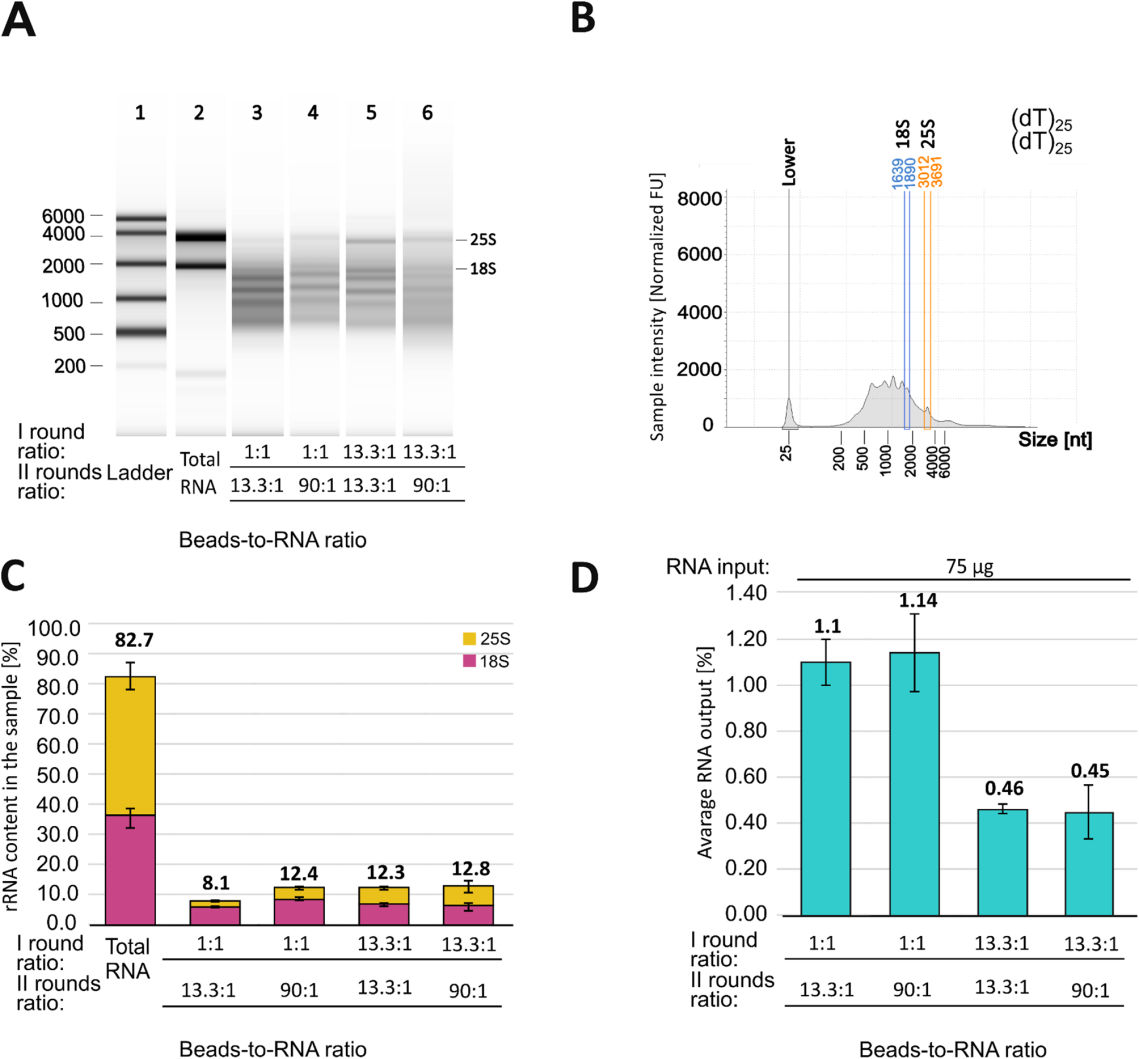
Results of a two-round mRNA enrichment using different (dT)_25_ beads-to-RNA ratios. (**A**) The electrophoretic results from the TapeStation platform. Lanes represent (1) RNA ladder; (2) total RNA input; (3–6) RNA samples after two rounds of mRNA enrichment. Applied beads-to-RNA ratios are provided below the lanes. (**B**) The representative electropherograms from the TapeStation platform. (**C**) Bar plot summary of rRNA content in the samples. (**D**) Bar plot summary of the average RNA output after mRNA enrichment. The RNA input for the first round was shown above the bars. A direct comparison of the results after the one- and two-round mRNA enrichment was shown in Supplementary Fig. 2.

Next, to further improve the results obtained with (dT)_25_ beads, we implemented an additional pre-purification step, resulting in two rounds of mRNA enrichment (Fig. 3). The first round of mRNA enrichment was performed with beads-to-RNA ratios of 13.3:1 and 1:1. All RNA from the first round was taken to the second round, where the beads-to-RNA ratio was adjusted to 13.3:1 or 90:1. Using the 1:1 ratio in the second round could significantly reduce the beads consumption. However, in this study, where the maximal tested RNA input was 75 μg and assuming a 3% RNA output (Fig. 2C), the volume of beads in the second round would be too small to operate correctly (only 0.5 μl). Nevertheless, it can be used instead for much higher total RNA inputs. For all samples, we found a substantial decrease in the intensity of the 18S and 25S signals, reaching levels between 8 and 12%, with the most favorable results observed in the 1:1—13.3:1 ratio combination (Fig. 3A–C, Supp. Figure 2). It represents a better improvement in rRNA depletion compared to the most efficient one-round mRNA enrichment with (dT)_25_, where rRNA still constituted approximately 20% of the output RNA sample (Fig. 2B). It is worth noting that the efficiency of the two-round procedure was very satisfactory, independent of applied beads-to-RNA ratios in each round. In addition to enhanced mRNA enrichment, this approach allows the use of much smaller amounts of magnetic beads than the application of one-round mRNA enrichment with the three highest tested (dT)_25_ beads-RNA ratios (Fig. 2B). Although the relative RNA output of the second round was notably higher than that of the first round (Supp. Figure 3), the improved efficiency of the two-round procedure was accompanied by lower final RNA output, ranging from 0.45 to 1.14% (Fig. 3D).

We also tested the application of the commercially available MAG and RiboMinus kits for the two-round mRNA enrichment. We considered the relation between the amounts of total RNA processed and reagent consumption as a crucial factor when choosing the approaches for the two-round procedure. Thus, the combinations we used were: MAG kit—RiboMinus kit, MAG kit—(dT)_25_ beads, (dT)_25_ beads—RiboMinus kit. We have decided against using the RiboMinus kit as the first step due to its low throughput and high cost (Table 1). The input cannot exceed 10 μg of total RNA per reaction, and as a result, the average output did not exceed 400 ng (Fig. 1D). This would necessitate the application of a significantly higher quantity of reagents to obtain enough RNA for the second round of mRNA enrichment. On the other hand, the MAG kit was not employed as the second step in the two-round mRNA enrichment. This particular kit uses optimized reagents, which have the benefit of significantly reducing the amount of magnetic beads required during the procedure. However, this also makes it challenging to work with RNA input lower than the recommended threshold of 30 μg due to an insufficient volume of magnetic beads in the tube.

**Fig. 4:**
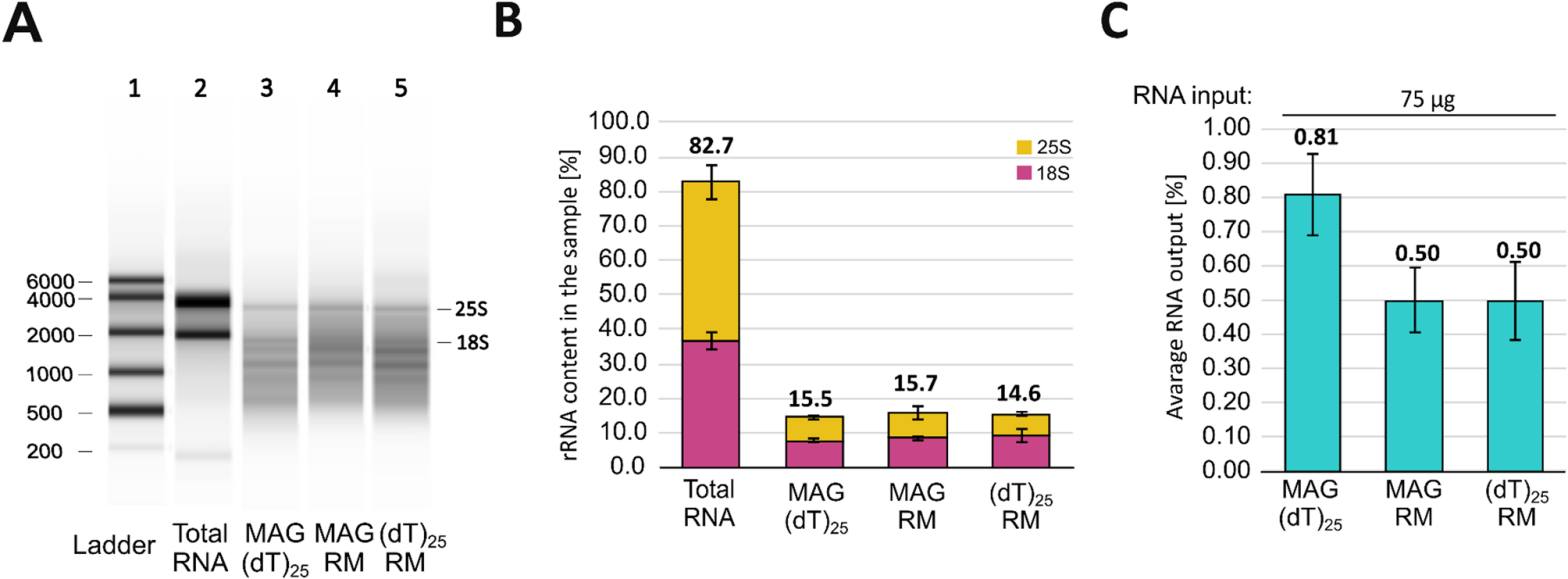
Results of the two-round mRNA enrichment with different method combinations. (**A**) The electrophoretic results from the TapeStation platform. Lanes represent (1) RNA ladder; (2) total RNA input; (3–5) RNA samples after two rounds of purification. The electropherogram results for all approaches are presented in Supplementary Fig. 1. (**B**) Bar plot summary of rRNA content in the final samples. (**C**) Bar plot summary of the final RNA output. A direct comparison of the results after the one- and two-round mRNA enrichment was shown in Supplementary Fig. 4.

For all tested combinations, we observed a significant decrease in the intensity of the 18S and 25S signals (Fig. 4A, B and Supp. Figure 1). However, the final rRNA content was slightly higher than for (dT)_25_ beads used twice, and ranged from 14.6% to 15.7%, depending on the combination used (Fig. 4B, Supp. Figure 4). The RNA output after two rounds was comparable and estimated at around 0.6% of the total RNA input (Fig. 4C). Considering cost and time requirements, the two-round mRNA enrichment with (dT)_25_ beads seems to be the optimal option, particularly when combined with reduced beads-to-RNA ratios. It also provides the best efficiency of rRNA depletion.

### NAI treatment does not impact mRNA enrichment with (dT)_25_ magnetic beads

**Fig. 5:**
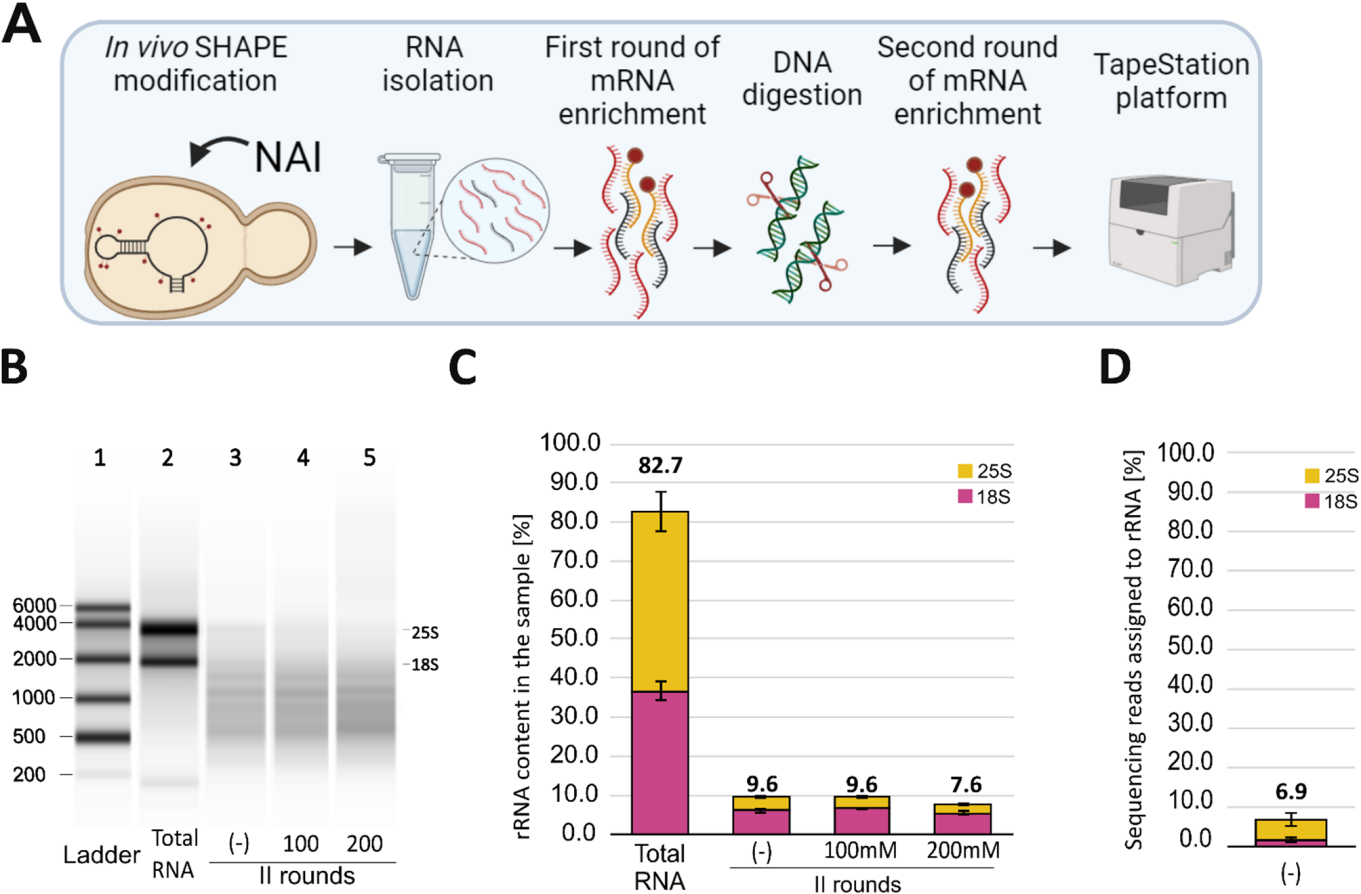
Results of the two-round mRNA enrichment after in vivo RNA modification with NAI. (**A**) Workflow of applied strategy. The applied (dT)_25_ beads to RNA ratios were 13.3:1 and 90:1. (**B**) The electrophoretic results from the TapeStation platform. Lanes represent (1) RNA ladder, (2) untreated total RNA; RNA samples after two rounds of purification with (dT)_25_ beads: (3) RNA treated with DMSO only, (4) 100 mM NAI, (5) 200 mM NAI. (**C**) Bar plot summary of rRNA content in the samples. (**D**) Bar plot representing the percentage of sequencing reads assigned to 18S and 25S rRNA sequences.

In the next step, we tested whether RNA modification with SHAPE reagents can affect the efficiency of the two-round mRNA enrichment strategy using the (dT)_25_ beads. SHAPE reagents are small-molecule electrophiles that measure RNA secondary structure by forming covalent adducts at the flexible ribose 2′-OH group^16^. To that end, we modified the yeast transcriptome with NAI in vivo, applied two rounds of mRNA enrichment with (dT)_25_ beads, and estimated the rRNA level using Tape Station (Fig. 5A). Although the DNA digestion step is typically performed during or immediately after RNA isolation, we performed it after the first round of mRNA enrichment. It allowed us to use a lower amount of DNase due to the reduction of non-polyadenylated RNA in the total RNA pool. Results from the TapeStation analysis indicated that this procedure resulted in even lower rRNA content in the final RNA samples (Fig. 5B, C). This improvement, however, was not a consequence of NAI modification, as the control sample treated with DMSO only (-) exhibited the same rRNA level as the sample treated with the standardly used NAI concentration (100 mM). We observed slightly lower rRNA content in the sample treated with 200 mM NAI. Thus, we claim that NAI treatment does not reduce mRNA enrichment efficiency. Moreover, the additional purification procedures after the DNA digestion step (e.g., RNA precipitation with ethanol or isopropanol) can contribute to efficiency improvement.

In SHAPE experiments, RNA modifications are typically detected during reverse transcription, followed by NG sequencing^17,18^. The presence of the NAI-derived adducts was confirmed by the electrophoretic analysis of dsDNA resulting from reverse transcription and second-strand synthesis (Supp. Figure 5). In the NAI-treated samples, there was a noticeable loss of longer products, which was due to a drop in the processivity of the reverse transcriptase caused by the adducts. In agreement with the TapeStation measurements, only 6.9% of sequencing reads were aligned to 18S and 25S rRNA sequences in the output sample (Fig. 5D). This supports the conclusion that the two-round procedure with (dT)_25_ beads is the optimal choice for mRNA enrichment in SHAPE experiments. These results also underline that the TapeStation system provides an excellent way to quickly and cheaply check the rRNA content in RNA samples, being an attractive alternative to NG sequencing.

## Discussion

In this study, we provided a comparative analysis of mRNA enrichment approaches in yeast total RNA samples. We evaluated the efficiency of mRNA enrichment conducted with two ready-to-use kits and oligo (dT) magnetic beads combined with custom buffers. We showed that a single round of enrichment in recommended conditions proved insufficient, with still approximately 50% of rRNA content in total RNA output, regardless of the approach employed. Surprisingly, neither of the commercial kits outperformed the (dT)_25_ beads regarding rRNA depletion. Therefore, we explored strategies to enhance mRNA enrichment efficiency. It involved testing different magnetic beads-to-RNA ratios and implementing a pre-treatment step, resulting in the two-round mRNA enrichment. Eventually, we found conditions that allowed for a much better reduction of rRNA content. Additionally, we present that the TapeStation capillary electrophoresis can be used for rRNA content assessment as an attractive alternative to NG sequencing.

Using (dT)_25_ magnetic beads, we showed that increasing the beads-to-RNA ratio significantly improved mRNA enrichment. Of note, decreasing the ratio from 13.3:1 to 1:1 has a negligible effect. Nevertheless, we achieved the highest efficiency in rRNA depletion by applying the two-round mRNA enrichment. Based on various combinations tested, we selected the two-round enrichment with (dT)_25_ magnetic beads, especially the combination of 1:1 and 13.3:1 ratios, as the best and most time- and cost-effective option (Table 1). The ratio lowering in the second round could also be considered for larger RNA inputs than those applied in this study. The two-round approach can reduce rRNA content to less than 10% in the final RNA sample. Such an efficient rRNA drop was not achieved in any single-step procedure, even at the highest tested beads-to-RNA ratio. However, the improved efficiency of mRNA enrichment was accompanied by a lower relative RNA output compared to the single-round procedure. On the other hand, the two-round approach requires significantly fewer magnetic beads to process the same amount of total RNA compared to the single-step procedures with the best mRNA enrichment efficiency. The additional economic advantage of the (dT)_25_ beads is the possibility of their regenerating and reusing in subsequent experiments.

Nevertheless, the choice of the mRNA enrichment strategy should also be influenced by the study’s goal and downstream analyses planned. Studies of RNA expression levels have demonstrated that the choice of rRNA depletion approach affects RNA-seq results^9^. Zhao et al. showed that methods based on poly(A) selection offer superior exonic coverage and improved accuracy in gene quantification^19^. On the other hand, rRNA depletion-based methods also capture nascent transcripts that have not yet undergone the polyadenylation process, significantly increasing the proportion of intronic sequences from pre-mature mRNAs (up to 50% of mapped reads). Consequently, more reads must be generated to achieve the same level of exonic coverage as when using the poly(A) selection approach. Similarly, Jaksik et al. demonstrated that poly(A) selection leads to better outcomes in analyzing gene expression and structural aberrations, while methods based on rRNA removal are more suitable for detecting various classes of RNAs, mutations, or polymorphisms^20^.

The persistence of some rRNA contamination, even after two rounds of mRNA enrichment, may be attributed to the substantial predominance of rRNA, constituting more than 80% of total RNA in yeast. It may pose a risk of non-specific rRNA binding to the oligo (dT) beads and, thus, their appearance in the RNA pool after elution. Moreover, although poly(A) tails are conventionally regarded as a distinguishing feature of mRNAs, polyadenylation of yeast rRNAs was also reported^21^. While the detected amount of polyadenylated rRNA is low, it becomes significant considering rRNA great abundance in the total RNA pool. A similar observation was also described for 18S and 28S rRNAs in human cells, where many post-transcriptionally added tails contained not only but primarily adenosines^22^. This phenomenon may contribute to rRNA ‘background’ when utilizing oligo (dT)-based approaches for mRNA selection. Our study omitted the presence of 5S rRNA in the total yeast RNA samples. According to the manufacturer, the RiboMinus kit does not target and deplete 5S rRNA. However, recent work demonstrates that applying the RiboMinus kit designed for bacteria allows the removal of approximately 40% of the 5S rRNA, as confirmed by the qPCR method^14^.

In the case of SHAPE experiments, a very high sequencing depth for each RNA nucleotide is needed to provide high-quality information about RNA secondary structure^23^. The simplest way to entirely circumvent the challenge of rRNA contamination involves employing gene- or region-specific primers in the modification detection during the reverse transcription process. However, non-specific primers must be used to obtain SHAPE data for the transcriptomes. Thus, finding the most efficient rRNA depletion strategy is still critical. Importantly, we demonstrated that RNA modification with one of the leading SHAPE reagents, NAI, does not impede the mRNA enrichment with (dT)_25_ magnetic beads.

To our knowledge, this is the first study to directly investigate the impact of NAI-based SHAPE probing on mRNA enrichment efficiency for *Saccharomyces cerevisiae*. The only paper that evaluated the impact of NAI on mRNA enrichment is Yang et al. 2020^24^. However, this analysis was performed for *Arabidopsis thaliana* total RNA*,* and the applied RNA probing method (CAP-STRUCTURE-seq) involved enzymatic digestion to selectively retain capped mRNAs rather than poly(A)-based enrichment or rRNA depletion. Therefore, although NAI was used in that study, its impact was not evaluated in the context of the standard protocols involving rRNA removal or mRNA enrichment. A lack of any significant interference with mRNA enrichment was shown for other RNA chemical probing reagents such as DMS, 1M7, or BzCN^25–27^. Moreover, these studies used different model organisms or cell lines (*Arabidopsis thaliana*, rice, HEK293 cells).

The effectiveness of mRNA enrichment may vary across different organisms. Factors such as variations in rRNA sequences, their processing, or the amount of polyadenylated non-coding rRNAs may contribute to differences in the performance of mRNA enrichment methods. Although caution should be exercised when extrapolating these results to other organisms, we believe that the general rules of the presented strategies will apply to the mRNA enrichment in most eukaryotic total RNA samples.

## Methods

### Media and growth conditions

Yeast strain *Saccharomyces cerevisiae* BY4741 was grown in 25 ml of yeast extract/peptone/dextrose (YPD) medium (BioShop) at 30 °C at 200 rpm. Cultures were diluted to an OD_600nm_ of 0.1 and grown in 300 ml of the YPD medium until OD_600nm_ of 0.8 was reached. Cells were collected by centrifugation at 4000×*g* for 5 min at 10 °C. Pellets were resuspended in PBS (BioShop) and centrifuged under the same conditions.

### RNA isolation

Yeast pellets were resuspended in 1 ml of lysis buffer (10 mM Tris–HCl, pH 8.5, 5 mM EDTA, 2% SDS, 2% 2-mercaptoethanol) and incubated at 83 °C for 20 min with constant shaking at 450 rpm. The samples were centrifuged at 12,300×*g* for 5 min at room temperature. The supernatants were transferred to fresh tubes and extracted twice in phenol (pH 8.0) and twice with phenol : chloroform (pH 4.5). RNA was recovered by LiCl precipitation overnight at –20 °C, followed by centrifugation at 16,100×*g* for 30 min at 4 °C. RNA pellets were washed twice with 70% ethanol and resuspended in 50 µl of water. RNA concentration and purity were measured by the NanoDrop 2000c spectrometer (Thermo Scientific).

### Applied mRNA enrichment methods

*RiboMinus™ Transcriptome Isolation Kit, yeast* (Invitrogen, cat. no. K155003). The procedure was conducted according to the manufacturer’s protocol. Briefly, 10 μg (0.5 µg/µl) of total yeast RNA was used as input. 250 µl (3000 µg) of RiboMinus™ Magnetic Beads were washed twice with 250 µl of RNase-free water, once with 250 µl of Hybridization Buffer (B10), and finally, resuspended in 100 µl of Hybridization Buffer (B10). Total RNA and 4 µl of RiboMinus™ Probe (100 pmol/µl) were added to 100 µl of Hybridization Buffer (B10), mixed, and incubated at 37 °C for 5 min, followed by placing it on ice. The mixture was then transferred to the prepared RiboMinus™ Magnetic Beads and incubated at 37 °C for 15 min (beads-to-RNA ratio of 300:1). The complex of rRNA with RiboMinus™ Probe was separated from the mixture using a magnetic separator, and the supernatant containing RNA was collected. The output RNA sample was then processed using the RiboMinus™ Concentration Module. Briefly, the obtained supernatant was mixed with 250 µl of Binding Buffer (L3) and 125 µl of 96% ethanol, loaded onto a concentration column, and washed with 200 µl of Wash Buffer (W5). The purified RNA was eluted with 15 µl of RNAse-free water.

*Poly(A)Purist™ MAG Kit* (Invitrogen, cat. no. AM1922). The procedure was conducted according to the manufacturer’s protocol. In brief, 75 μg of total yeast RNA (600 μg/ml) was used for a single enrichment procedure. An equivalent mass of beads (75 μg in 7.5 μl) was washed twice with 75 μl of Wash Solution 1. Then, the RNA sample was mixed with one volume of 2X Binding Solution and added to the beads (beads-to-RNA ratio of 1:1). The mixture was incubated at 75 °C for 5 min, followed by incubation at room temperature for 60 min. The beads were then captured, and the supernatant was removed. The beads were washed twice with Wash Solution 1 and 2 in a volume equal to the initial RNA sample, followed by elution in RNA Storage Solution prewarmed to 80 °C. The eluted poly(A) RNA was precipitated with 5 M ammonium acetate, glycogen, and ethanol at − 80 °C for 30 min. The purified RNA was dissolved in 20 µl of RNAse-free water.

*Oligo (dT)*_*25*_* Magnetic Beads* (New England Biolabs, cat. no. S1419S)*.* The procedure was conducted according to the protocol by Green and Sambrook^15^. The total yeast RNA input was 75 µg (750 ng/µl). Briefly, 100 µl of binding buffer (0.5 M LiCl, 0.1 M Tris–HCl, pH 8.0, 10 mM EDTA, 1% SDS, 5 mM DTT) was added to the total RNA, and the samples were heated for 5 min at 70 °C. Then, the solution was cooled on ice for 1 min. 200 µl (1000 µg) of magnetic beads were washed with 100 µl of binding buffer, followed by resuspension in another 100 µl of the same buffer. RNA solution was mixed with the beads (beads-to-RNA ratio of 13.3:1) and rotated continuously for 5 min at room temperature. In the next step, the beads were washed three times with 1 ml of washing buffer (0.15 M LiCl, 10 mM Tris–HCl, pH 8.0, 1 mM EDTA, 0.1% SDS). The mRNA captured on the beads was incubated with 30 µl of elution buffer (10 mM Tris–HCl, pH 8.0) for 2 min at 80 °C. The elution step was repeated three times.

### Testing diverse magnetic beads-to-RNA ratios

The experiment involved an mRNA enrichment procedure in six different ratios of the oligo (dT)_25_ beads to total RNA, namely 1:1, 3:1, 6:1, 25:1, 50:1, and 125:1. This was achieved by adjusting either the RNA or beads amount. In detail, for 1:1, 3:1, 6:1, and 25:1 ratios, we used an RNA input of 75 µg (in 100 µl) with an increasing amount of beads (75, 225, 450, and 1875 µg, respectively). For both 50:1 and 125:1 ratios, we used RNA input of 5 µg and 2 µg (in 100 µl) with the appropriate amount of beads (250 and 100 µg for 50:1 ratio, 625 and 250 µg for 125:1 ratio, respectively). For these two ratios, we reduced the volume of washing buffer to 500 µl, and due to low RNA concentration, samples were precipitated with 5 M ammonium acetate, glycogen, and ethanol at − 80 °C for 30 min, followed by resuspension in 16 µl of water.

### The second round of mRNA enrichment

In the experiment involving two rounds of mRNA enrichment with oligo (dT)_25_ magnetic beads, the total RNA output from the first round was taken to the second round. The beads amount for the second round was adjusted to achieve the ratio of 13.3:1 or 90:1. The overall procedure was conducted as described above for the two highest beads-to-RNA ratios.

The total RNA output from the purification with the Poly(A)Purist™ MAG kit or with oligo (dT)_25_ magnetic beads was taken for the second round of mRNA enrichment using different approaches. Samples were processed with the RiboMinus™ kit or oligo (dT)_25_ magnetic beads. The procedure for oligo (dT)_25_ magnetic beads was modified as described above for the second round. RNA was precipitated by 5 M ammonium acetate, glycogen, and ethanol at − 80 °C for 30 min. The final product was resuspended in 16 µl of water.

### In vivo NAI modification of yeast RNA and mRNA enrichment

Yeast pellet from 150 ml of the culture was resuspended in 300 µl PBS, divided into two equal samples, and then PBS was added to each sample to a total volume of 360 µl. For in vivo RNA modification, cells were treated with 40 µl of 2-methylnicotinic acid imidazolide (NAI, Sigma Aldrich) diluted in DMSO (the final NAI concentration of 100 mM or 200 mM). In the control sample, cells were treated with DMSO alone. Samples were incubated at 30 °C for 20 min with gentle agitation. RNA modification reaction was quenched by adding 400 µl of 1 M DTT (BioShop) and gentle rotation. Then, cells were collected at 4000×*g* for 4 min at room temperature, washed with 500 µl of PBS, and centrifuged under the same conditions.

After in vivo SHAPE modification, the total RNA isolation step was performed as described above. The first round of mRNA enrichment was performed using 75 µg of total RNA and 1000 µg of oligo (dT)_25_ magnetic beads (beads-to-RNA ratio of 13.3:1). After that, DNA digestion was performed (Turbo DNAse, Invitrogen) for 30 min at 37 °C. RNA was extracted with phenol : chloroform (pH 4.5) and then precipitated with ammonium acetate (5 M), glycogen, and ethanol at − 80 °C for 30 min. RNA was recovered by centrifugation at 16,100×*g* for 30 min at 4 °C. RNA pellets were washed once with 100 µl of 70% ethanol and resuspended in 50 µl of water. The second round of mRNA enrichment was conducted using 250 µg of oligo (dT)_25_ magnetic beads (beads-to-RNA ratio of 90:1). Then, RNA was precipitated by 5 M ammonium acetate, glycogen, and ethanol at − 80 °C for 30 min. The final product was resuspended in 16 µl of water.

### RNA quality assessment

The integrity of RNA was measured on a Qubit fluorometer using RNA IQ Assay Kit (Invitrogen).

### rRNA depletion efficiency assessment by TapeStation capillary electrophoresis

mRNA enrichment efficiency was assessed after the first and second round using the TapeStation 4150 (Agilent Technologies) electrophoresis platform and High Sensitivity RNA ScreenTape. Signal intensity from TapeStation tapes was analyzed using TapeStation Analysis software 5.1. In brief, RNA lengths were distinguished based on the band positions in the electrophoretic profiles, and the global scaling and automated background correction for all analyzed tapes were applied. Percentages of rRNA content were calculated and normalized using ImageQuantTL 10.2 software. In brief, the intensity of bands and the corresponding electropherogram peaks representing 25S and 18S rRNAs were quantified, and the component quantity was calculated relative to the intensity of the entire sample. No additional background cutoff was applied in this step. All experiments were performed in triplicate.

### rRNA depletion efficiency assessment by NG sequencing

RNA samples treated with DMSO only were further processed according to the SHAPE-MaP protocol^23^. In brief, the RNA sample was incubated with random nonamer primers (200 ng/µl, New England Biolabs) for 5 min at 65 °C. After cooling to 4 °C, 8 µl of 2.5 × MaP Buffer (125 mM Tris–HCl, pH 8.0, 187.5 mM KCl, 25 mM DTT, 1.25 mM dNTPs, 15 mM MnCl_2_) and 1 µl of SUPERase·In™ RNase Inhibitor (20U/ µl Invitrogen) was added, followed by incubation for 2 min at 25 °C. Finally, 1 µl of reverse transcriptase enzyme (SuperScript II, Invitrogen) was added, and the solution was incubated at 25 °C for 10 min, at 42 °C for 3 h, at 75 °C for 5 min, and cooled at 4 °C. After that, EDTA was added (to the final concentration of 6 mM), followed by incubation for 5 min at room temperature and the addition of MgCl_2_ (to the final concentration of 6 mM). Second-strand synthesis was conducted using NEBNext® Ultra™ II Non-Directional RNA Second-Strand Synthesis Module (New England Biolabs) at 16 °C for 3 h. The obtained dsDNA was purified using GeneMATRIX PCR/DNA Clean-Up Purification Kit (Euryx) and eluted with 50 µl water. In the next step, samples were precipitated by ammonium acetate (5 M), glycogen, and ethanol at − 80 °C for 30 min and recovered by centrifugation at 16,100×*g* for 30 min at 4 °C. dsDNA pellet was washed once with 100 µl of 70% ethanol and resuspended in 15 µl of water. The final dsDNA amount was measured using Qubit high-sensitivity dsDNA assay (Invitrogen). The following steps of library preparation and sequencing were performed by Novogene (UK). Sequencing was performed on the Illumina platform (NovaSeq) using a 2 × 150 bp read configuration, resulting in a total data of 60–115 Gb obtained in three replicates, with about 89% of the base calls having a quality score above Q30. Sequencing reads alignment to 18S and 25S ribosomal RNAs was performed using Bowtie2, implemented in ShapeMapper 2^28,29^.

## Electronic supplementary material

Below is the link to the electronic supplementary material.

Supplementary Material 1

## Data Availability

Raw sequencing data can be obtained from https://www.ncbi.nlm.nih.gov/bioproject/ and can be accessed under accession code PRJNA1134665. Other data generated or analyzed during this study are included in this published article and its supplementary file.
