# Supplementary material for: A comparative analysis of mRNA enrichment strategies and guidance for improving their efficiency
Source: Sci Rep. 2025 May 23;15:17890. doi: 10.1038/s41598-025-02082-z (PMC12102362; doi:10.1038/s41598-025-02082-z)
Supplement: Supplementary file 1 — Supplementary Material 1 [file 41598_2025_2082_MOESM1_ESM.pdf]

SUPPLEMENTARY FIGURES

A comparative analysis of mRNA enrichment strategies and guidance for improving their efficiency.

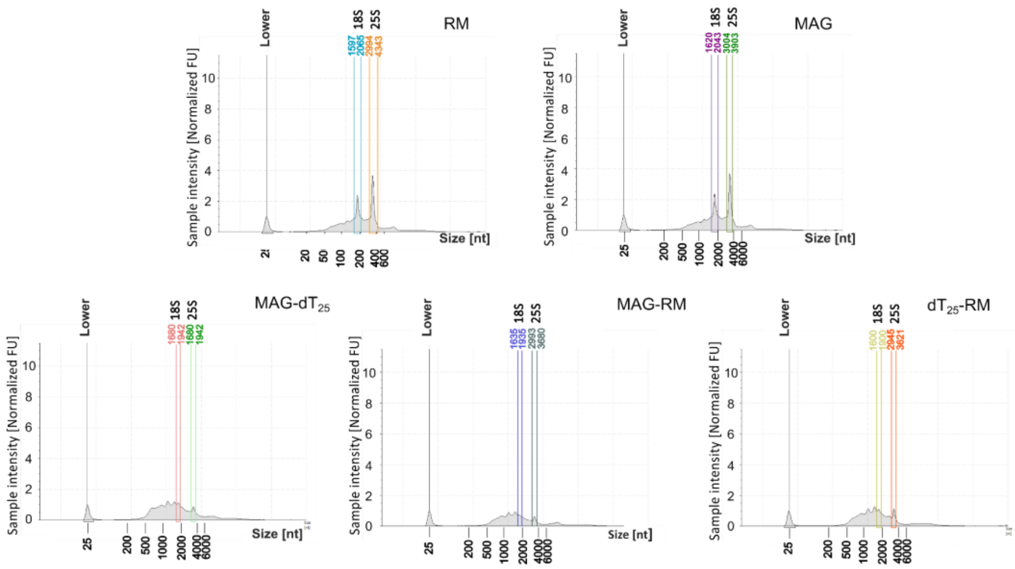

Supplementary Fig. 1. The electropherograms are generated by the TapeStation platform. The graphs represent samples after a single round of mRNA enrichment (RiboMinus and MAG kits) and after two rounds of mRNA enrichment in different combinations (MAG kit - $(dT)_{25}$ beads, MAG kit - RiboMinus kit, $(dT)_{25}$ beads – RiboMinus kit).

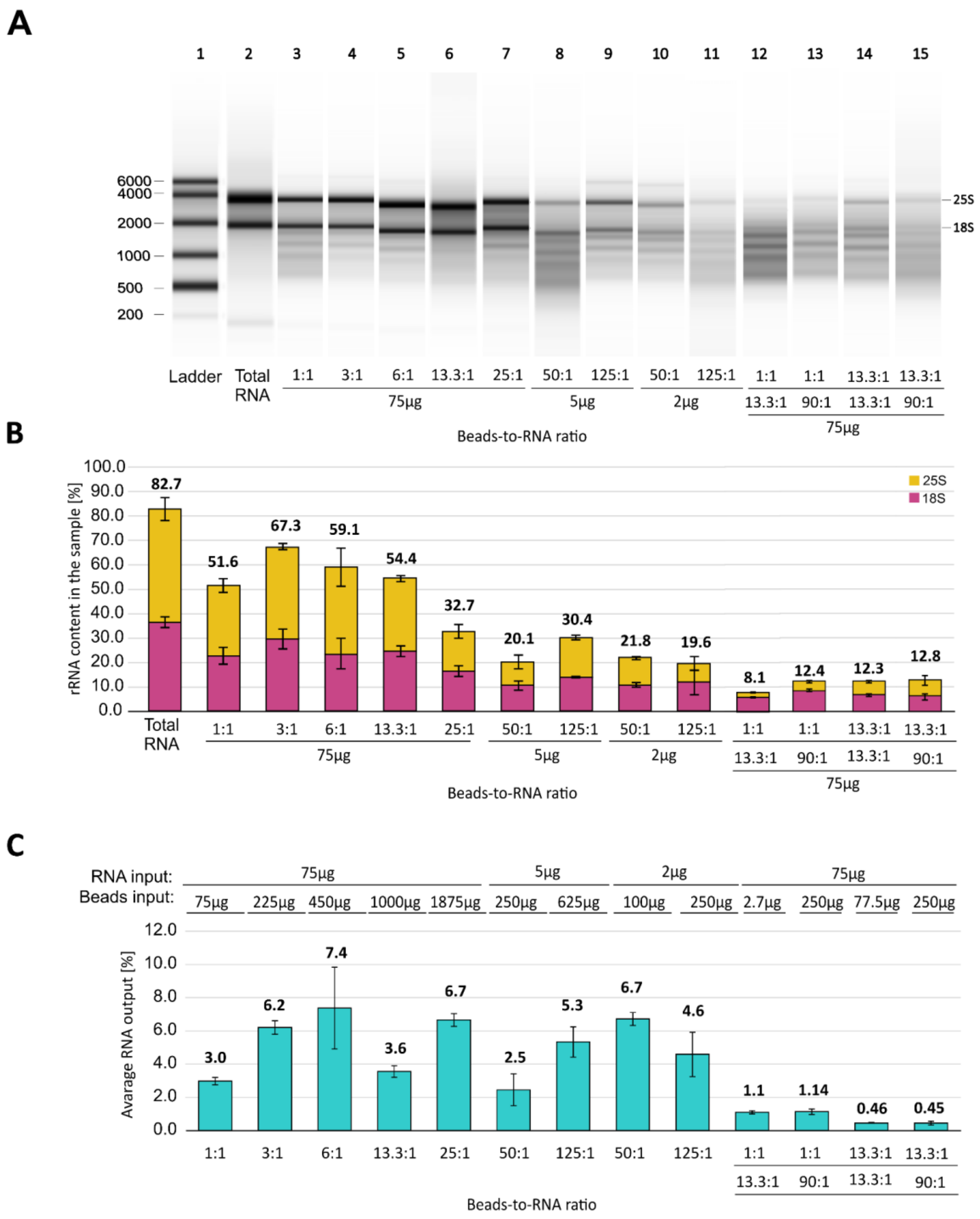

Supplementary Fig. 2. Results of a one- and two-round mRNA enrichment using different $(dT)_{25}$ beads-to-RNA ratios. **(A)** The electrophoretic results from the TapeStation platform. Lanes represent (1) RNA ladder; (2) total RNA input; (3-11) RNA samples after one round of mRNA enrichment; (12–15) RNA samples after two rounds of mRNA enrichment. Applied beads-to-RNA ratios are provided below the lanes. **(B)** Bar plot summary of rRNA content in the samples. **(C)** Bar plot summary of the average RNA output after mRNA enrichment. The RNA input for the first round was shown above the bars.

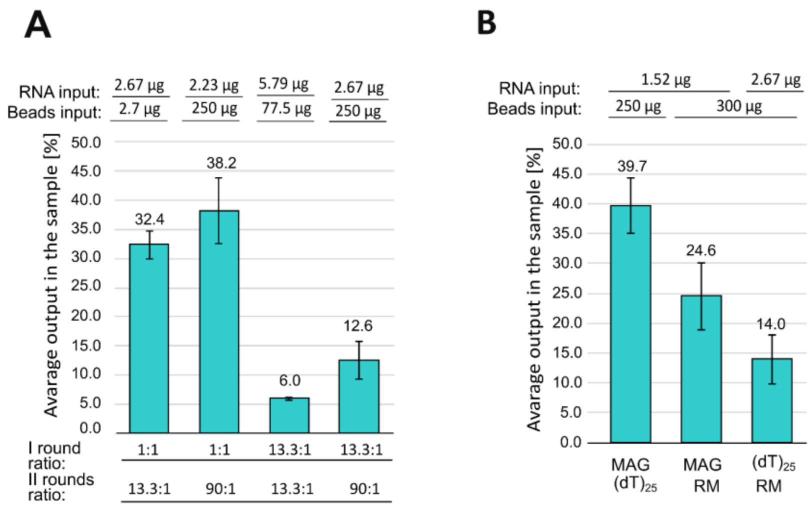

Supplementary Fig. 3. The bar plot summary of the average relative RNA output after the second round of mRNA enrichment **(A)** using $(dT)_{25}$ beads in different beads-to-RNA ratios and **(B)** using different method combinations. The RNA and bead inputs are shown above the corresponding bars.

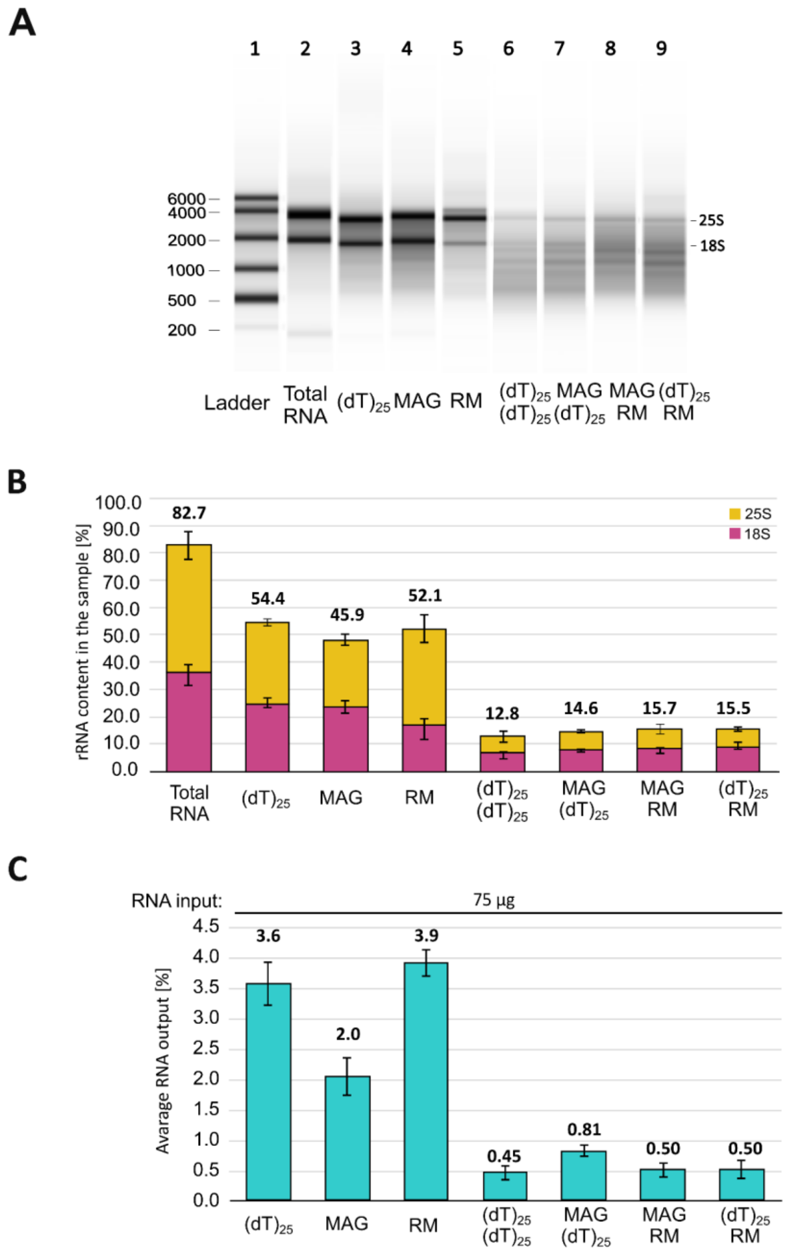

Supplementary Fig. 4. Results of the one- and two-round mRNA enrichment with different method combinations. **(A)** The electrophoretic results from the TapeStation platform. Lanes represent (1) RNA ladder; (2) total RNA input; (3–5) RNA samples after one round of purification; (6–9) RNA samples after two rounds of purification. **(B)** Bar plot summary of rRNA content in the final samples. **(C)** Bar plot summary of the final RNA output.

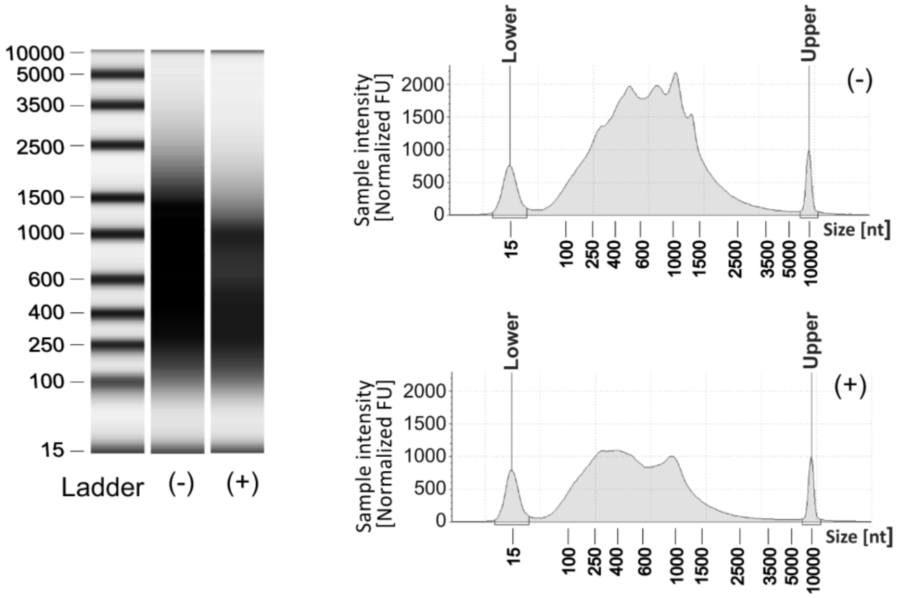

Supplementary Fig. 5. The electrophoretic result from the TapeStation platform for dsDNA samples obtained after the SHAPE-MaP procedure combined with two rounds of mRNA enrichment with $(dT)_{25}$ beads. RNA samples were treated with DMSO only (-) or 100 mM NAI (+).
